# Influence of area-level social vulnerability on all-cause pneumonia, all-cause acute otitis media, and invasive pneumococcal disease incidence among Medicaid-enrolled children

**DOI:** 10.1186/s41479-025-00186-8

**Published:** 2025-12-25

**Authors:** Salini Mohanty, Michael Barna, Kelsie Cassell, Nicole Cossrow, Peter C. Fiduccia, Esther Smith-Howell, Valina C. McGuinn, Alyssa Evans, Aparna Keshaviah, Priya Shanmugam, Saumya Chatrath, Constance Delannoy, Kristen A. Feemster, Lisa Weissburg, Jelena Zurovac

**Affiliations:** 1 Merck & Co.,Inc, P.O. Box 2000, 126 East Lincoln Ave, Rahway, NJ 07065 USA; 2 Mathematica, Inc., Princeton, NJ USA

**Keywords:** Pediatric pneumonia, All-cause pneumonia, Acute otitis media, Invasive pneumococcal disease, Claims data, Medicaid, Pneumococcal conjugate vaccine, United States

## Abstract

**Background:**

The incidence of pneumococcal disease differs based on demographic and clinical factors, yet the impact of area-level social determinants of health, particularly for children, remains less understood. We characterized the relationship between individual and area-level social vulnerability and incidence of all-cause pneumonia (ACP), acute otitis media (AOM), and invasive pneumococcal disease (IPD) among children across the US.

**Methods:**

Using a retrospective observational design, we measured disease incidence among children 18 years and younger covered by Medicaid, using claims from 2017 through 2019. We measured social vulnerability using quintiles of the county-level Minority Health Social Vulnerability Index (MHSVI) and its six subthemes (Socioeconomic Status; Household Composition and Disability; Minority Status and Language; Housing Type and Transportation; Health Care Infrastructure and Access; Medical Vulnerability). After calculating county-level ACP, AOM, and IPD incidence rates overall and among counties in each MHSVI quintile, we used Poisson regression to characterize the relationship between social vulnerability and disease incidence, controlling for confounding by child demographic characteristics. We analyzed this relationship overall and within age and race/ethnicity subgroups.

**Results:**

Across 38.1 million children, ACP, AOM, and IPD incidence rates were 1,767, 16,486, and 3.3 per 100,000 person-years, respectively. ACP and AOM incidence rates were lower among children in the most versus least socially vulnerable counties (unadjusted incidence rate ratio = 0.89 and 0.75, respectively; both *P* < 0.0001); differences were attenuated but remained statistically significant after adjusting for child demographics. The direction of the relationship between ACP and AOM incidence and vulnerability varied by type of vulnerability: incidence was lower in counties that were most (versus least) vulnerable based on Minority Status and Language or Housing Type and Transportation, but higher in those most vulnerable based on Household Characteristics or Disability and Medical Vulnerability. Differences in IPD incidence between the most versus least vulnerable counties were generally non-significant, due to the low overall incidence.

**Conclusions:**

Incidence of AOM and ACP in children varies significantly by demographic characteristics and county-level medical and non-medical social determinants of health. Social vulnerability may be useful to identify factors associated with disparities in pneumococcal disease and develop targeted interventions to reduce them.

**Supplementary information:**

The online version contains supplementary material available at 10.1186/s41479-025-00186-8.

## Background

*Streptococcus pneumoniae*, a Gram-positive bacterium, is a leading cause of bacterial pneumonia in children [1] and is responsible for both invasive pneumococcal diseases (IPD; including bacteremia and meningitis) and non-invasive diseases (including non-bacteremic pneumococcal pneumonia and acute otitis media [AOM]) [2]. In the United States (US), an estimated 1.5 million children develop all-cause pneumonia (ACP) annually, including pneumococcal, bacterial, and viral cases, with healthcare costs for both insurers and patients totaling $3.6 billion annually [3]. Further, ACP is one of the most common causes of hospitalization among children [4]. *S. pneumoniae* is one of the main bacterial causes of AOM, a leading reason young children receive outpatient care and antibiotics [5, 6]. Within the first three years of life, 60% of US children have at least one AOM episode [7], resulting in an estimated 16 million cases of AOM in children each year with healthcare costs of $4.3 billion annually [3].

Pneumococcal disease burden varies by demographic and socioeconomic characteristics. For instance, US children who are male, non-Hispanic White, have a family history of AOM, or attend daycare, are at higher risk of AOM episodes [7]. In addition to individual risk factors, area-level social determinants of health (SDOH)—including nonmedical factors such as housing, transportation, and healthcare access—also influence disease burden. Children in rural and low-income areas in the US, where healthcare access is limited due to affordability and availability, have lower uptake of the pediatric pneumococcal vaccine [8] and higher rates of pneumococcal disease [9, 10].

Our study aimed to characterize the relationship between area-level social vulnerability and ACP, AOM, and IPD incidence among children 18 and younger enrolled in Medicaid from 2017 through 2019. In 2018, Medicaid covered over 46 million children—more than a third of all children in the US [11]. This study builds upon our previous work, which characterized the relationship between area-level social vulnerability and disease burden, including ACP and IPD, among adults in Medicaid [12]. Our findings can inform state and local officials by enhancing their understanding of pneumococcal disease burden in children to support development of targeted interventions to better safeguard highly vulnerable families and children [13, 14].

## Methods

### Data sources

To examine the link between area-level social vulnerability and disease burden, we conducted a retrospective cohort study, integrating Medicaid claims data on ACP, AOM, and IPD incidence with county-level social vulnerability data from the Centers for Disease Control and Prevention (CDC).

We analyzed Medicaid claims from January 1, 2017, through December 31, 2019, excluding data from 2020 onward to avoid changes in disease incidence due to the COVID-19 pandemic. Specifically, we leveraged enrollment files and medical claims from the Transformed Medicaid Statistical Information System Analytic File (TAF) claims database maintained by CMS to (a) identify enrollees meeting our eligibility criteria (described in the Study population section below); (b) define enrollee subgroups; and (c) calculate disease incidence.

County-level social vulnerability data for all 50 US states and the District of Columbia came from the Minority Health Social Vulnerability Index (MHSVI), a composite measure of vulnerability released in 2021 by the CDC [15]. The MHSVI incorporates 34 social indicators, derived from CDC health outcomes data, US Census Bureau demographics, and other publicly available data. These indicators are grouped into six theme-level indices: Socioeconomic Status, Household Composition and Disability, Minority Status and Language, Housing Type and Transportation, Health Care Infrastructure and Access, and Medical Vulnerability (Appendix Table A1). Both the composite index and theme indices are scored from 0 to 1, with higher values indicating higher social vulnerability.

To measure urbanicity, we used the 2013 Rural-Urban Continuum Codes [16] from the 2022–2023 Area Health Resource File, as follows: urban (metropolitan), suburban (non-metropolitan with more than 2,500 people), or rural (fewer than 2,500 people) (Appendix Table A2).

### Study population

The cohort included children aged 18 and younger enrolled in Medicaid for at least six continuous months during the study period. Enrollment length and continuity restrictions were applied to minimize bias from incomplete disease event data due to enrollment gaps or shorter enrollment periods. Children were excluded if they were dually eligible for Medicare and Medicaid because their disease episodes may not be fully captured in Medicaid claims, with Medicare serving as their primary payer. Further, we excluded children receiving long-term care services during the study period, as disease tracking for this group would be unreliable. Finally, we excluded children residing in states with unreliable Medicaid claims data (Appendix Table A3), and in counties lacking MHSVI data.

### Disease incidence measures

Disease incidence was based on International Classification of Diseases, Tenth Revision (ICD-10) diagnosis codes for non-invasive ACP and all-cause AOM (caused by *S. pneumoniae* or other pathogens), and IPD (see Appendix Table A4 for included codes, based on [17] and [3]). We chose to focus on ACP and all-cause AOM because the current methods to detect bacterial pathogens in non-invasive disease are limited [18]. Children with IPD, ACP, or AOM episodes were identified in each calendar year using inpatient and outpatient claims. For each enrollee in the cohort, we grouped claims into disease episodes, using the first claim with an ACP, AOM, or IPD code. If multiple diagnoses appeared on claims with the same date, we prioritized based on severity: IPD, then ACP, then AOM. To prevent overcounting, we required a 30-day gap between IPD and ACP episodes; that is, multiple episodes occurring within 30 days of an index claim were considered a single IPD or ACP episode. Similarly, we required a 14-day gap between AOM episodes. These adjustments reduced the likelihood that claims for the same disease episode were erroneously counted as separate episodes. We also excluded episodes occurring within a child’s first month of Medicaid enrollment, as these could reflect an ongoing episode. Our approach to defining disease episodes aligns with prior studies on pneumococcal disease burden [17, 19].

To calculate disease incidence, we summed the number of distinct disease episodes for each of ACP, AOM, and IPD across all children and years, and we divided the result by the sum of years each child contributed during the study period minus the number of disease episodes. We report incidence per 100,000 person-years (PY).

### Social vulnerability measures

To evaluate how ACP, AOM, and IPD incidence rates varied by area-level social vulnerability, we grouped counties into quintiles of MHSVI, which ranged from Q1 = least vulnerable (0 ≤ MHSVI < 0.2) to Q5 = most vulnerable (0.8 ≤ MHSVI ≤ 1). We then calculated disease incidence by MHSVI quintile by aggregating child-level data across counties assigned to each quintile, based on each child’s most recent county of residence.

### Statistical analyses

We explored how incidence varied spatially, by mapping county-level ACP, AOM, and IPD incidence rates. As part of an exploratory spatial analysis, we calculated the Moran’s I coefficient to measure spatial autocorrelation (Appendix Table A5). In the maps, we did not report estimates for counties with fewer than 90 person-years to reduce noise in the estimates, that is, to avoid comparisons between counties where disease incidence estimates might reflect random variation due to small population counts.

We examined the relationship between area-level vulnerability and disease incidence using Poisson regression. The outcome was the count of disease episodes per child during the study period, with an offset term accounting for PYs contributed during the study period. Unadjusted models included MHSVI quintile indicators only, whereas adjusted models controlled for confounders, including: age group (less than 1 year, 1 year, 2–4 years, 5–18 years, based on younger children’s higher risk for pneumococcal disease [Centers for Disease Control and Prevention (CDC), 2024a]), sex (male, female), race and ethnicity (non-Hispanic White, non-Hispanic Black, Hispanic, Asian, other, missing), and urbanicity (urban, suburban, rural).

Results are shown as incidence rate ratios (IRRs), calculated as the disease incidence in the most vulnerable counties divided by the incidence in the least vulnerable counties (MHSVI Q5/Q1).

We assessed model fit before and after covariate adjustment using Akaike’s Information Criterion, where lower values indicate better fit. To evaluate potential confounding, we examined how the difference in disease incidence between the most and least vulnerable counties changed between the unadjusted and adjusted models.

Analyses were repeated within demographic subgroups (age group and race and ethnicity). We also analyzed the relationship between disease incidence and MHSVI themes.

All analyses were performed in SAS (version 9.3 or later) and Stata (version 18 or later), with statistical significance defined as *P* < 0.05 (two-sided, unadjusted).

## Results

Of over 50 million children enrolled in Medicaid during the study period, 4.5 million were excluded for not meeting eligibility criteria, and 7.3 million were excluded due to residing in states with data quality concerns or in counties lacking MHSVI data, leaving 38.1 million children in the analysis (Appendix Table A6). Slightly over half of children (51.1%) were male and 67.9% were aged 5–18. A vast majority (84.3%) lived in urban areas (Table 1) while 30.6% were non-Hispanic White, 25.4% Hispanic, and 16.6% non-Hispanic Black.

**Table 1:** Characteristics of children in Medicaid cohort (2017–2019)

Group	Number of children in group	Percentage	ACP incidence per 100,000 PY	AOM incidence per 100,000 PY	IPD incidence per 100,000 PY
**Overall**	38,106,592	100.0	1,767	16,486	3.3
**Gender**^**a**^					
Male	19,472,396	51.1	1,846	17,048	3.8
Female	18,620,966	48.9	1,685	15,901	2.8
**Age group (years)**^**b**^					
< 1	4,463,679	11.7	4,046	49,991	11.3
1	2,345,947	6.2	3,918	50,593	7.6
2–4	6,398,734	16.8	2,915	28,112	4.3
5–18	25,870,005	67.9	1,074	7,370	1.9
**Race and ethnicity**					
White, non-Hispanic	11,668,956	30.6	1,858	20,224	3.0
Black, non-Hispanic	6,308,212	16.6	1,515	11,048	3.5
Hispanic	9,678,079	25.4	1,538	14,194	2.5
Asian	1,292,228	3.4	1,935	8,957	2.7
Other	916,553	2.4	2,099	16,736	4.9
Missing	8,242,564	21.6	2,062	19,571	4.5
**Urbanicity**					
Urban	32,138,776	84.3	1,731	15,446	3.3
Suburban	5,372,087	14.1	1,958	22,020	3.4
Rural	595,729	1.6	1,947	22,290	2.6

ACP = all-cause pneumonia; AOM = acute otitis media; IPD = invasive pneumococcal disease; PY = person years

^a^ Rows for gender do not sum to overall number of enrollees, because those with missing information (*N* = 13,230) were excluded from the table

^b^ The sum of children across age groups is larger than the total number of children in the cohort because we partitioned each child’s study period such that their outcomes count towards the age group when they were within the corresponding age range

### All-cause pneumonia

Among the 38.1 million children in the cohort, the unadjusted ACP incidence was 1,767 per 100,000 PY (Table 1). ACP incidence was higher among younger children (4,046 and 3,918 per 100,000 PY for those under age 1 and 1 year old, respectively), Asian or other race and ethnicity (1,935 and 2,099 per 100,000 PY, respectively), and those living in suburban and rural counties (1,958 and 1,947 per 100,000 PY, respectively).

**Fig. 1:**
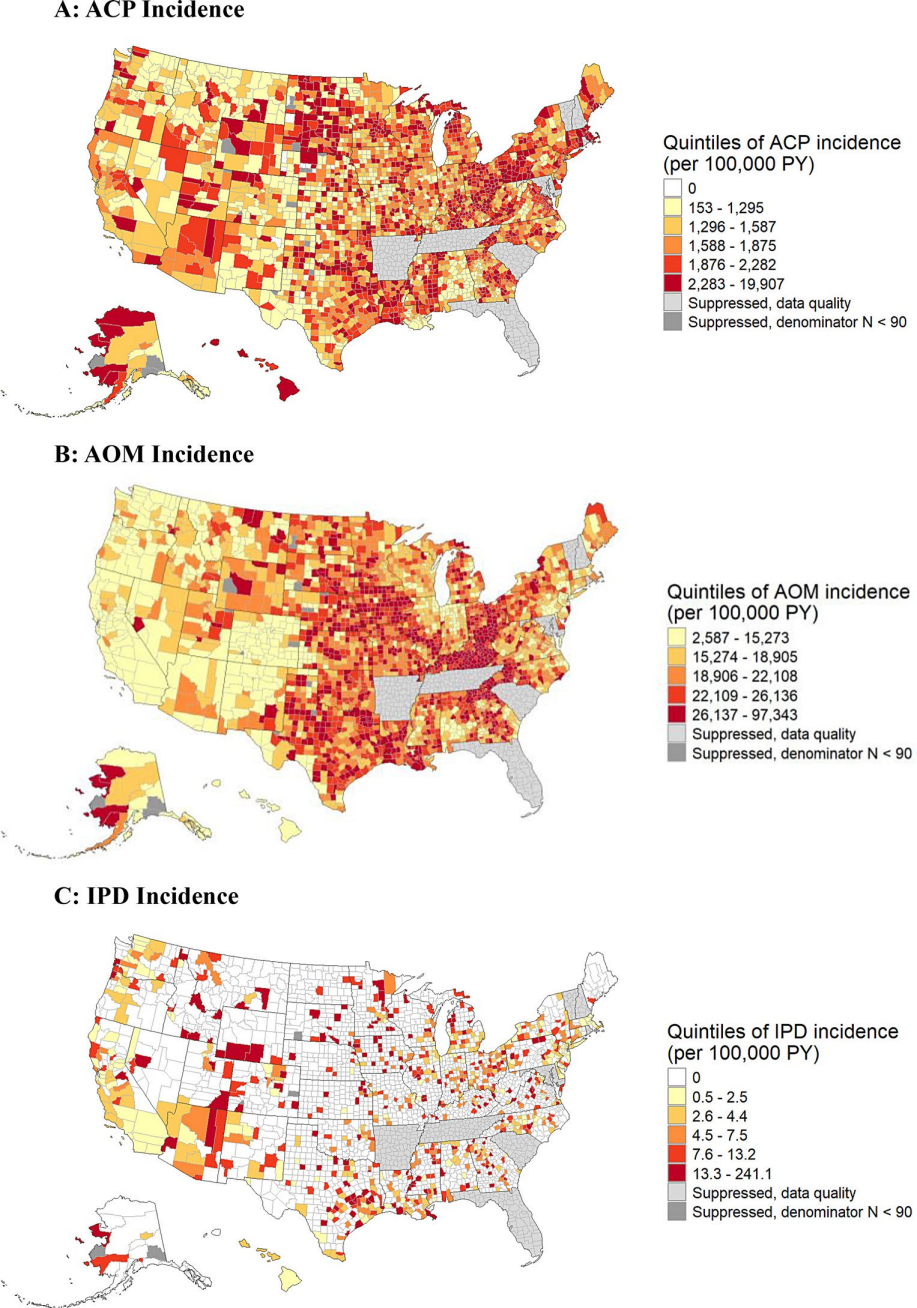
Unadjusted county-level ACP, AOM, and IPD incidence among children in Medicaid cohort (2017–2019). **A**: ACP incidence. **B**: AOM incidence. **C**: IPD incidence. Notes: The choropleth maps show US counties classified by disease incidence rate quintile. Data from Arkansas, Florida, Maryland, New Hampshire, Rhode Island, South Carolina, Tennessee, and Vermont were suppressed due to data quality below desired standards. We also suppressed data for counties with fewer than 90 person-years (*N* = 12). Very small and very large disease incidence rates are plausible for counties with small populations. State borders are in black lines and county borders are in gray. ACP = all-cause pneumonia; AOM = acute otitis media; PY = person-years

County-level ACP incidence rates ranged from 0 to 19,907 per 100,000 PY (Fig. 1A), and exhibited spatial clustering, indicating that neighboring counties had more similar disease rates than counties farther apart (Appendix Table A5). We observed a cluster of counties in the mid-Atlantic and in New England with higher ACP incidence, and generally lower ACP incidence in Western counties.

Based on unadjusted analyses, ACP incidence overall was significantly lower among children in the most versus least vulnerable counties (IRR = 0.89, *P* < 0.0001) (Table 2; Appendix Table A7). This pattern was consistent across almost all age groups (except for children under age 1, who had similar ACP incidence regardless of county vulnerability). This pattern also persisted across most race and ethnicity groups, with two exceptions. Children classified as other race had comparable ACP incidence across different county vulnerability levels while Asian children had 35% higher ACP incidence if they lived in the most versus least vulnerable counties (IRR = 1.35, *P* < 0.0001).

**Table 2:** Unadjusted difference in ACP, AOM, and IPD incidence among children in Medicaid cohort in the most versus least socially vulnerable counties (2017–2019)

Group	Incidence rate per 100,000 PY		Unadjusted IRR (Q5/Q1)	95% confidence interval around IRR	IRR *P*-value
	MHSVI Q1 counties	MHSVI Q5 counties			
**ACP**					
**Overall**	1,888	1,688	0.89	(0.886, 0.902)	< 0.0001
**Age group (years)**					
< 1	4,129	4,195	1.02	(0.993, 1.039)	0.17
1	4,045	3,897	0.96	(0.938, 0.989)	0.005
2–4	2,949	2,783	0.94	(0.928, 0.961)	< 0.0001
5–18	1,228	977	0.80	(0.784, 0.806)	< 0.0001
**Race and ethnicity**					
White, non-Hispanic	1,828	1,790	0.98	(0.967, 0.992)	0.001
Black, non-Hispanic	1,812	1,493	0.82	(0.788, 0.861)	< 0.0001
Hispanic	1,619	1,489	0.92	(0.894, 0.947)	< 0.0001
Asian	1,440	1,947	1.35	(1.258, 1.454)	< 0.0001
Other	2,162	2,225	1.03	(0.973, 1.088)	0.31
**AOM**					
**Overall**	20,653	15,432	0.75	(0.745, 0.749)	< 0.0001
**Age group (years)**					
< 1	63,750	47,167	0.74	(0.736, 0.744)	< 0.0001
1	63,870	47,186	0.74	(0.734, 0.744)	< 0.0001
2–4	35,025	26,266	0.75	(0.746, 0.754)	< 0.0001
5–18	9,297	6,865	0.74	(0.735, 0.742)	< 0.0001
**Race and ethnicity**					
White, non-Hispanic	19,778	19,815	1.00	(0.998, 1.006)	0.35
Black, non-Hispanic	13,723	10,341	0.75	(0.742, 0.766)	< 0.0001
Hispanic	18,570	14,147	0.76	(0.755, 0.768)	< 0.0001
Asian	13,000	8,877	0.68	(0.666, 0.700)	< 0.0001
Other	18,056	17,958	0.99	(0.976, 1.014)	0.58
**IPD**					
**Overall**	3.9	3.4	0.87	(0.709, 1.058)	0.16
**Age group (years)**					
< 1	13.9	11.6	0.83	(0.562, 1.232)	0.36
1	9.5	8.1	0.85	(0.492, 1.460)	0.55
2–4	4.2	4.4	1.04	(0.652, 1.651)	0.88
5–18	2.4	1.9	0.81	(0.592, 1.096)	0.17
**Race and ethnicity**					
White, non-Hispanic	4.1	2.8	0.69	(0.525, 0.917)	0.010
Black, non-Hispanic	1.8	3.8	2.14	(0.532, 8.592)	0.28
Hispanic	3.2	2.5	0.79	(0.410, 1.539)	0.50
Asian	7.6	2.9	0.37	(0.133, 1.054)	0.06
Other	1.7	6.9	4.18	(0.580, 30.126)	0.16

ACP = all-cause pneumonia; AOM = acute otitis media; IRR = incidence rate ratio; MHSVI = Minority Health Social Vulnerability Index; PY = person-years; Q1 = least vulnerable MHSVI quintile; Q5 = most vulnerable MHSVI quintile.

After controlling for child-level characteristics, overall and among most subgroups, ACP incidence remained lower in the most versus least vulnerable counties, though differences were attenuated (adjusted IRR = 0.97 overall, *P* < 0.0001) (Table 3; Appendix Table A8). In a few subgroups, regression adjustment increased the differences between the most and least vulnerable counties, including among infants (adjusted IRR = 1.17 versus unadjusted IRR = 1.02), non-Hispanic White children (adjusted IRR = 0.95 versus unadjusted IRR = 0.98), and Hispanic children (adjusted IRR = 0.88 vs unadjusted IRR = 0.92). Model fit also improved with regression adjustment for child-level characteristics, both overall and within each subgroup (Appendix Table A9).

**Table 3:** Adjusted difference in ACP, AOM, and IPD incidence among children in Medicaid cohort in the most versus least socially vulnerable counties (2017–2019)

Group	Number of children in regression	Regression-adjusted IRR (Q5/Q1)	95% confidence interval around IRR	IRR *P*-value
**ACP**				
**Overall**	38,093,362	0.97	(0.957, 0.975)	< 0.0001
**Age group (years)**^**a**^				
< 1	4,452,021	1.17	(1.141, 1.196)	< 0.0001
1	2,345,152	1.05	(1.023, 1.080)	0.0003
2–4	6,398,422	0.98	(0.963, 0.999)	0.04
5–18	25,869,518	0.87	(0.859, 0.883)	< 0.0001
**Race and ethnicity**				
White, non-Hispanic	11,664,065	0.95	(0.935, 0.960)	< 0.0001
Black, non-Hispanic	6,303,386	0.82	(0.787, 0.860)	< 0.0001
Hispanic	9,677,359	0.88	(0.855, 0.909)	< 0.0001
Asian	1,292,061	1.27	(1.181, 1.370)	< 0.0001
Other	916,336	1.05	(0.989, 1.107)	0.12
**AOM**				
**Overall**	38,093,362	0.93	(0.930, 0.936)	< 0.0001
**Age group (years)**^**a**^				
< 1	4,452,021	0.92	(0.912, 0.923)	< 0.0001
1	2,345,152	0.91	(0.908, 0.921)	< 0.0001
2–4	6,398,422	0.93	(0.922, 0.931)	< 0.0001
5–18	25,869,518	0.97	(0.964, 0.974)	< 0.0001
**Race and ethnicity**				
White, non-Hispanic	11,664,065	0.99	(0.989, 0.996)	0.0001
Black, non-Hispanic	6,303,386	0.75	(0.737, 0.761)	< 0.0001
Hispanic	9,677,359	0.85	(0.847, 0.862)	< 0.0001
Asian	1,292,061	0.79	(0.772, 0.812)	< 0.0001
Other	916,336	1.05	(1.029, 1.070)	< 0.0001
**IPD**				
**Overall**	38,093,362	0.86	(0.701, 1.057)	0.15
**Age group (years)**^**a**^				
< 1	4,452,021	0.86	(0.576, 1.294)	0.48
1	2,345,152	0.94	(0.537, 1.654)	0.84
2–4	6,398,422	0.95	(0.593, 1.534)	0.85
5–18	25,869,518	0.80	(0.581, 1.097)	0.16
**Race and ethnicity**				
White, non-Hispanic	11,664,065	0.66	(0.495, 0.873)	0.004
Black, non-Hispanic	6,303,386	1.84	(0.459, 7.410)	0.39
Hispanic	9,677,359	0.73	(0.366, 1.455)	0.02
Asian	1,292,061	0.27	(0.092, 0.784)	0.37
Other	916,336	4.45	(0.611, 32.351)	0.14

Note: Regression adjustment controlled for age group, race and ethnicity, gender, urbanicity, and MHSVI quintile

ACP = all-cause pneumonia; AOM = acute otitis media; IRR = incidence rate ratio; MHSVI = Minority Health Social Vulnerability Index; Q1 = least vulnerable MHSVI quintile; Q5 = most vulnerable MHSVI quintile

^a^ The sum of children across age groups is larger than the total number of children in the cohort because we partitioned each child’s study period such that their outcomes count towards the age group when they were within the corresponding age range

**Fig. 2:**
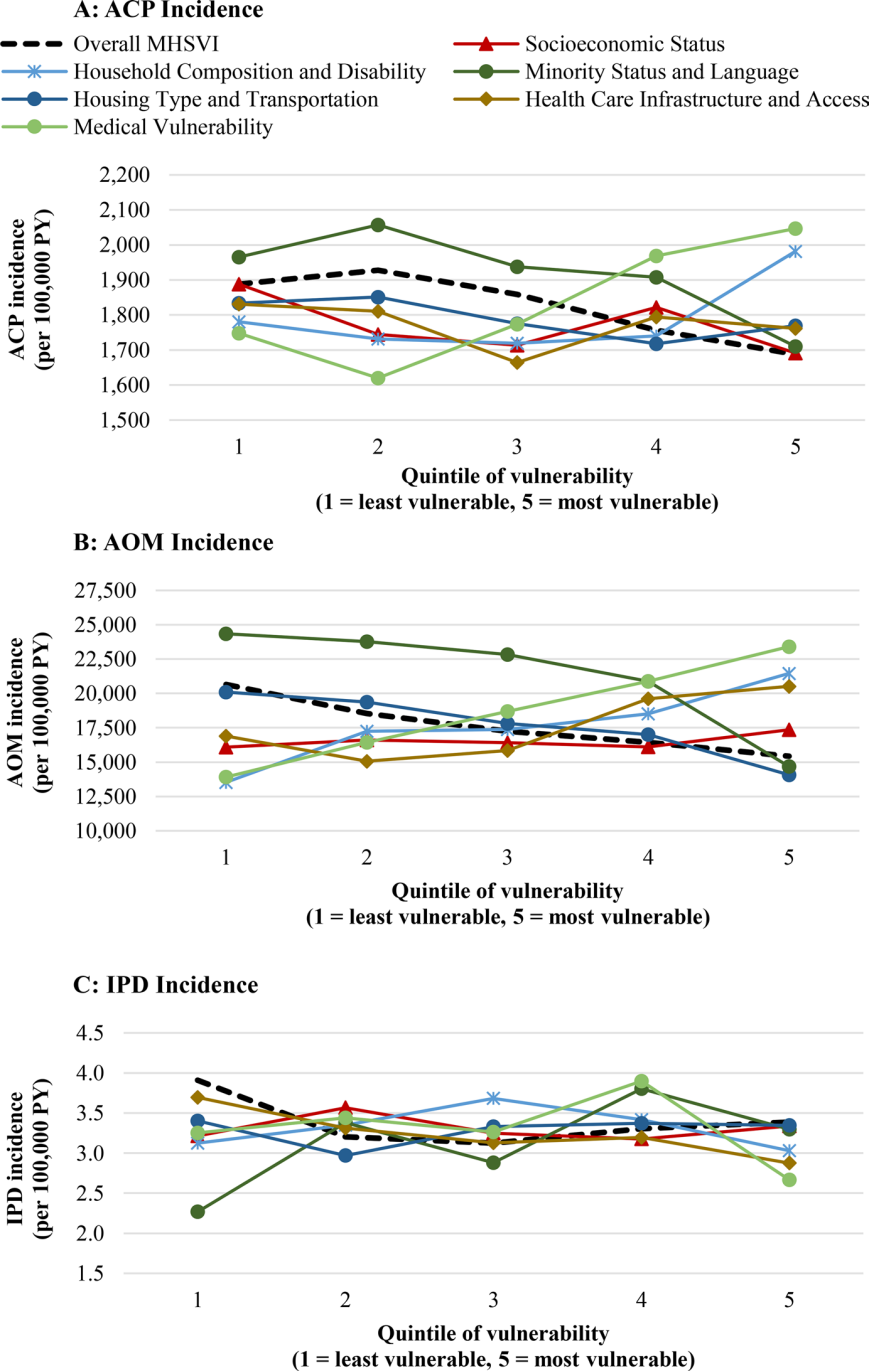
Unadjusted ACP, AOM, and IPD incidence among children in Medicaid cohort, by MHSVI overall and theme quintiles (2017–2019). **A**: ACP incidence. **B**: AOM incidence. **C**: IPD incidence. Notes: the graphs plot the ACP, AOM, and IPD incidence in counties in each MHSVI quintile, with one line per MHSVI index (the overall index and six theme indices). See Appendix table A10 for the underlying data. ACP = all-cause pneumonia; AOM = acute otitis media; IPD = invasive pneumococcal pneumonia; MHSVI = minority health social vulnerability Index; PY = person-years

The relationship between social vulnerability and ACP incidence varied by vulnerability type (Fig. 2A; Appendix Table A9). When vulnerability was defined by Minority Status and Language or Socioeconomic Status, findings were similar to overall MHSVI results, with more vulnerable counties having significantly lower ACP incidence (IRR = 0.87, *P* < 0.0001; IRR = 0.90, *P* = 0.02, respectively). In contrast, when vulnerability was based on Medical Vulnerability or Household Composition and Disability, the relationship was reversed, with more vulnerable counties showing higher ACP incidence (IRR = 1.17, *P* < 0.0001; IRR = 1.11, *P* = 0.02, respectively). No significant relationship was observed when vulnerability was defined by Housing Type and Transportation or on Health Care Infrastructure and Access.

### Acute otitis media

The overall unadjusted AOM incidence was 16,486 per 100,000 PY (Table 1), with higher rates among younger children (49,991 and 50,593 per 100,000 PY for children under age 1 and 1 year old, respectively) and non-Hispanic White children (20,224 per 100,000 PY). County-level AOM incidence ranged from 2,587 to 97,343 per 1,000 PY, with evidence of spatial clustering. Similar to ACP, AOM incidence was higher in clusters of counties in the mid-Atlantic and in New England, and lower in the West and Pacific Northwest (Fig. 1B; Appendix Table A5).

Unadjusted AOM incidence was generally lower in counties with higher social vulnerability, based on the overall MHSVI index (IRR = 0.75, *P* < 0.0001) (Table 2; Appendix Table A7). This pattern persisted across age groups. For race and ethnicity subgroups, AOM incidence was lower in the most versus least vulnerable counties among non-Hispanic Black, Hispanic, and Asian children (IRR = 0.75, 0.76, and 0.68, respectively, *P* < 0.0001 for all three). However, no significant differences were observed for non-Hispanic White or other race and ethnicity children (IRR = 1.00 and 0.99, respectively).

After adjusting for child characteristics, AOM incidence remained significantly lower in the most versus least vulnerable counties overall and in subgroup analyses, but differences were attenuated (adjusted IRR = 0.93, *P* < 0.0001) (Table 3; Appendix Table A9). Among non-Hispanic White children and those classified as Other race and ethnicity, differences in AOM incidence between most versus least vulnerable counties were statistically significant after regression adjustment (adjusted IRR = 0.99, *p* = 0.0001; adjusted IRR = 1.05, *p* < 0.0001, respectively). As with ACP incidence, adjusting for child characteristics improved model fit overall and across subgroup analyses (Appendix Table A8).

The relationship between social vulnerability and AOM incidence followed a similar pattern as for ACP incidence (Fig. 2B; Appendix Table A9). Namely, counties with greater vulnerability in Minority Status and Language or Housing Type and Transportation had lower AOM incidence (IRR = 0.60 and 0.70, respectively, *P* < 0.0001 for both). In contrast, counties with greater vulnerability in Medical Vulnerability or Household Composition and Disability had higher AOM incidence (IRR = 1.68 and 1.59, respectively, *P* < 0.0001 for both).

### Invasive pneumococcal disease

The overall unadjusted IPD incidence was 3.3 per 100,000 PY (Table 1), with higher rates among younger children (11.3 and 7.6 per 100,000 PY for children under age 1 and 1 year old, respectively) and those classified as other race and ethnicity (4.9 per 100,000 PY). County-level IPD incidence ranged from 0 to 241 per 100,000 PY, with no IPD cases observed among children in many counties (Fig. 1C).

IPD incidence was lower in the most vulnerable counties compared to the least vulnerable counties, but the difference was not statistically significant, at least in part due to small population counts (IRR = 0.87, *P* = 0.16) (Table 2; Appendix Table A7). Regression adjustment for demographic characteristics improved model fit, but did not meaningfully alter the difference in IPD incidence, either overall (IRR = 0.86, *P* = 0.15), or within most subgroups, where differences largely remained non-significant (Table 3; Appendix Table A8). Finally, we observed no clear relationship between IPD incidence by different types of vulnerability, based on the MHSVI themes (Fig. 2C; Appendix Table A9).

## Discussion

In this large-scale analysis of Medicaid-enrolled children, we examined how ACP, AOM, and IPD incidence rates varied by area-level social vulnerability. Overall, ACP and AOM incidence rates were significantly lower among children in the most vulnerable counties than the least vulnerable counties, whereas IPD incidence did not significantly vary by area-level social vulnerability.

Our estimates of ACP, AOM, and IPD incidence, together with the relatively higher incidence we observed among younger children and males, align with prior research [17, 20, 21]. With respect to area-level vulnerability, the pattern of lower ACP incidence among children in the most vulnerable counties mirrors results from our previous work for vaccination uptake [22] among adult Medicaid enrollees. Research by Qian and Rehkopf [23] found the same pattern for otitis media among commercially insured children in the US, who were less likely to be treated for recurrent otitis media, suppurative otitis media, and tympanostomy tube insertion if they lived in more vulnerable areas (based on the social deprivation index or social vulnerability index).

The Minority Status and Language theme was the primary driver of the association between greater social vulnerability and lower ACP and AOM incidence, with Housing Type and Transportation also contributing for AOM. These findings are consistent with prior research among adult Medicaid enrollees [12]. For Minority Status and Language, the association may reflect health literacy. Yin et al. [24] found that children from US households with lower health literacy were more likely to encounter barriers to reaching providers after hours and difficulty traveling to a primary care clinic. Similarly, children in non-English-speaking households in the US are likely to face comparable difficulties in accessing healthcare [25].

It is well documented that transportation barriers are associated with reduced access to healthcare [26]. A key indicator within the Housing Type and Transportation theme is lack of vehicle access, which is associated with higher odds of missed appointments in urban, low-income settings and among families reliant on public transportation [26]. In an urban pediatric setting, Wallace et al. [27] found that longer travel times by bus or car from low-income neighborhoods were associated with higher odds of missed appointments. Crowding, another component of the Housing Type and Transportation theme, has been linked to higher risk of carrying *S. pneumoniae* [28]. Thus, the lower incidence we observed among children with greater housing and transportation vulnerability in our predominantly urban cohort may reflect reduced access to care and underdiagnosis, rather than a truly lower disease burden. This interpretation aligns with prior work showing that socially advantaged children appear at greater risk for AOM because they are more likely to receive treatment, whereas socially disadvantaged children are less likely to receive treatment but more likely to be hospitalized for severe complications of undertreated otitis media [23]. Transportation barriers and low health literacy are more likely to affect access to care for AOM than for ACP, given that AOM is more likely to resolve spontaneously. However, given that both ACP and AOM include viral pathogens as causes, it is possible that transportation barriers and health literacy are a part of the explanation for lower disease incidence for both outcomes, as our results and the literature suggest.

Further, we found higher AOM incidence among non-Hispanic White children than other racial and ethnic groups, consistent with previous studies in the US [7, 29]. Gerber et al. [29] suggested that the higher incidence was not related to biologic factors, but rather to differences in non-Hispanic White parents’ expectations about antibiotic treatment for AOM, which may have resulted in more frequent diagnosis of AOM.

Differences in ACP and AOM incidence between the most versus least socially vulnerable counties were smaller after adjusting for individual characteristics, similar to our previous research for adults in Medicaid [11]. This attenuation suggests that part of the variation in incidence across counties is explained by the demographic composition of the enrolled population, that is, socially vulnerable individuals often live in more vulnerable areas [12]. Our models intentionally adjusted only for age, sex, race/ethnicity, and urbanicity, given that some risk factors for pneumococcal disease (such as asthma) may themselves be shaped by area-level medical vulnerability and are differentially captured in claims. Consistent with our study aim to describe area-level patterns rather than isolate a causal effect, we preserved potential pathways through which social context and healthcare access influence *measured* incidence. Although the Medical Vulnerability theme incorporates comorbidities such as chronic respiratory disease, it does not capture all risk factors for pneumococcal disease. Future work should continue to monitor pneumococcal vaccination uptake in relation to area-level social vulnerability. Our related analyses of adult Medicaid beneficiaries demonstrated that disparities in uptake were already present in 2016–2019 [22] and became more pronounced during the COVID-19 pandemic [30].

Our study has several limitations. First, our findings may not fully generalize to the broader Medicaid population due to eligibility restrictions, including continuous enrollment, exclusion of dual enrollees and long-term care recipients, and the limitation to states with high-quality data. Additionally, requiring continuous enrollment may have skewed the study population toward children in families with more stable income and healthcare access, potentially underrepresenting those in families with fluctuating incomes. However, enrollment and disenrollment among Medicaid children is less common than in adults [31]. Data quality issues also present limitations. Despite excluding states with poor-quality Medicaid data, some data inconsistencies may have remained and introduced measurement error in disease incidence across geographic areas. Additionally, using a hierarchical approach to define disease episodes may have slightly underestimated ACP and AOM incidence. The small number of IPD episodes in the Medicaid population affected the IPD analysis, for which results were largely non-significant. Also, comparisons of incidence across counties should be interpreted cautiously given small counts in some counties. Furthermore, race and ethnicity subgroup findings should be considered exploratory, given the high rates of missing race/ethnicity data in Medicaid records. Finally, our regression results may have been affected by unmeasured confounders and mediators, such as individual health behaviors. We observed spatial autocorrelation but did not account for it in the analysis because our goal was to estimate differences in disease burden between more and less vulnerable areas—rather than to isolate the effect of MHSVI-dependent vulnerability, after accounting for all other geographically dependent factors. Additionally, given the large number of statistical comparisons, there was some risk of spurious statistical significance. We chose not to adjust the Type I error rate to avoid obscuring potentially meaningful relationships in this novel analysis.

## Conclusions

Our analyses revealed lower ACP and AOM incidence in the most versus least socially vulnerable counties in the US among Medicaid-enrolled children. Because claims-based incidence reflects both true disease burden and patterns of healthcare use, these findings should be interpreted in the context of known barriers to diagnosis and care among vulnerable populations. Our results describe geographic and vulnerability-related patterns in observed disease incidence, which may help inform future research and guide interventions to better safeguard highly vulnerable children.

## Electronic supplementary material

Below is the link to the electronic supplementary material.

supplementary material 1

## Data Availability

The data for Medicaid enrollees are available under restricted access due to CMS requirements. Our data use agreement (DUA) with CMS prevents us from sharing data, so they cannot be made publicly available. MHSVI data are publicly available as of March 13, 2025, at https://minorityhealth.hhs.gov/minority-health-svi. Area Health Resource Files are publicly available, at https://data.hrsa.gov/data/download.

## Abbreviations

ACP: All-cause pneumonia
AOM: Acute otitis media
CDC: Centers for Disease Control and Prevention
CMS: Centers for Medicare & Medicaid Services
COVID-19: Coronavirus disease, 2019
ICD-10: International Classification of Diseases, Tenth Revision
IPD: Invasive pneumococcal disease
IRR: Incidence rate ratio
MHSVI: Minority Health Social Vulnerability Index
PY: Person-years
Q: MHSVI quintile
SDOH: Social determinants of health
TAF: Transformed Medicaid Statistical Information System Analytic Files
US: United States
