# Supplementary material for: Influence of area-level social vulnerability on all-cause pneumonia, all-cause acute otitis media, and invasive pneumococcal disease incidence among Medicaid-enrolled children
Source: Pneumonia (Nathan). 2025 Dec 25;17:35. doi: 10.1186/s41479-025-00186-8 (PMC12739848; doi:10.1186/s41479-025-00186-8)
Supplement: Supplementary file 1 — supplementary material 1 [file 41479_2025_186_MOESM1_ESM.docx]

# Influence of area-level social vulnerability on all-cause pneumonia, all-cause acute otitis media, and invasive pneumococcal disease incidence among Medicaid-enrolled children *Supplemental appendix tables and figures*

Salini Mohanty^1^, Michael Barna^2^, Kelsie Cassell^1^, Nicole Cossrow^1^, Peter C. Fiduccia^1^, Esther Smith-Howell ^1^, Valina C. McGuinn^1^, Alyssa Evans^2^, Aparna Keshaviah^2^, Priya Shanmugam^2^, Saumya Chatrath^2^, Constance Delannoy^2^, Kristen A. Feemster^1^, Lisa Weissburg^2^, and

Jelena Zurovac^2^

^1^ Merck & Co., Inc., Rahway, NJ, USA

^2^ Mathematica, Inc., Princeton, NJ, USA

**Table of contents**

[Table A1. Themes of Minority Health Social Vulnerability Index 2](#_Toc194612049)

[Table A2. County-level urbanization classification using 2013 Rural-Urban Continuum Codes 3](#_Toc194612050)

[Table A3. States removed from Medicaid cohort analyses due to data quality issues 4](#_Toc194612051)

[Table A4. Diagnosis codes used to identify pneumococcal disease 5](#_Toc194612052)

[Table A5. Moran’s I to assess spatial autocorrelation in county-level disease incidence rates among Medicaid children 7](#_Toc194612053)

[Table A6. Study cohort construction – number of Medicaid children at each stage of cohort creation 7](#_Toc194612054)

[Table A7. Unadjusted ACP, AOM, and IPD incidence among children in Medicaid cohort, by group and county-level social vulnerability quintile, overall MHSVI (2017–2019) 9](#_Toc194612055)

[Table A8. Difference in ACP, AOM, and IPD incidence among children in the Medicaid cohort residing in the most versus least socially vulnerable counties (2017–2019) 10](#_Toc194612056)

[Table A9. Unadjusted ACP, AOM, and IPD incidence among children in the Medicaid cohort, by MHSVI quintile and theme (2017–2019) 11](#_Toc194612057)

## **Table A1. Themes of Minority Health Social Vulnerability Index**

| **Overall Minority Health Social Vulnerability Index** | | | | | |
| --- | --- | --- | --- | --- | --- |
| **Socioeconomic Status** | **Household Composition  and Disability** | **Minority Status  and Language** | **Housing Type  and Transportation** | **Health Care Infrastructure  and Access** | **Medical  Vulnerability** |
| - Persons below 150% of Federal Poverty Line - Unemployed - Housing cost burden**^†^** - No high school diploma | - ≥65 Years - ≤17 Years - Civilian with  a disability - Single-parent households* | - Hispanic or Latino - Black or African American - Asian - American Indian  or Alaska Native - Native Hawaiian  or Pacific Islander - Other Race - Two or More Races - Foreign language: speaking English less than “very well” | - Multi-unit structures** - Mobile homes** - Crowding (more people than rooms)* - No vehicle* - Persons living in group quarters | - Hospitals** - Urgent care clinics** - Pharmacies** - Primary care physicians** - No health insurance | - Cardiovascular disease mortality - Chronic respiratory disease prevalence - Obesity prevalence - Diabetes prevalence - Internet access |
| Themes 1-4 are based on ACS data | | | | Based on geospatial data from RX Open, HIFLD, and the AHRF | Based on data from IHME, CDC’s Diabetes Surveillance System and the Interactive Atlas of Heart Disease and Stroke |

Note: The themes and variables used to construct those themes are based on the 2021 version of the scale.

*Measured at the county-level; **Measured at household level. All other variables measured at the individual-level.

ACS = American Community Survey; AHRF = Area Health Resources File; HIFLD = Homeland Infrastructure Foundation-Level Data; IHME = the Institute for Health Metrics and Evaluation.

## **Table A2. County-level urbanization classification using 2013 Rural-Urban Continuum Codes**

| **RUCC Description** | **Classification** |
| --- | --- |
| Counties in metro areas of 1 million population or more | Urban |
| Counties in metro areas of 250,000 to 1 million population | Urban |
| Counties in metro areas of fewer than 250,000 population | Urban |
| Urban population of 20,000 or more, adjacent to a metro area | Suburban |
| Urban population of 20,000 or more, not adjacent to a metro area | Suburban |
| Urban population of 2,500 to 19,999, adjacent to a metro area | Suburban |
| Urban population of 2,500 to 19,999, not adjacent to a metro area | Suburban |
| Completely rural or less than 2,500 urban population, adjacent to a metro area | Rural |
| Completely rural or less than 2,500 urban population, not adjacent to a metro area | Rural |

Source: USDA Economic Research Service (2025).

## **Table A3. States removed from Medicaid cohort analyses due to data quality issues**

| State | Data quality concerns in DQ Atlas in one or more years of measurement period (2017–2019) | | | Notes |
| --- | --- | --- | --- | --- |
|  | Identifying those for whom vaccine is recommended | Determining county of residence | Identifying disease episodes |  |
| Florida | **✓** |  | **✓** | Low other services user rate |
| Maryland | **✓** |  | **✓** | Low diagnosis code capture in inpatient claims |
| New Hampshire | **✓** |  | **✓** | Low inpatient user rate |
| Rhode Island | **✓** | **✓** |  | Low ZIP code capture and low inpatient user rate |
| Tennessee | **✓** |  | **✓** | Low diagnosis code capture in inpatient claims |
| Vermont |  | **✓** |  | Low ZIP code capture |

Note: Despite passing all data quality checks, we also excluded Arkansas and South Carolina from our analysis due to implausibly low disease incidence rates that were quite different from bordering states.

Each state collects and reports Medicaid data, but several states have known data quality issues. State variation in data quality could impact estimates of disparities in disease incidence between geographic areas. To ensure accuracy, we assessed Medicaid data quality by state and excluded states with critical data quality issues.

**Methods for DQ Atlas investigation.^[[1]](#footnote-2)^** We omitted states from the analyses for which the DQ Atlas (a tool that reports Medicaid data quality) classified data fields critical for the analysis as “high concern” or “unusable” in at least one year within the study period (2017–2019).

1. **County of residence.** To estimate county-level disease incidence, we must determine the county of residence for each enrollee.
2. **Diagnosis code.** We use the diagnosis codes in inpatient (IP) and other services (OT^[[2]](#footnote-3)^) TAF files to classify individuals into risk categories – at risk or high risk – as well as to identify disease episodes
3. **Proportion of service users.** We also consider proportions of Medicaid enrollees with at least one claim record in the IP and OT files.

*Additional contextual factors.* We also considered additional state-specific information, which led us to include one state—Georgia—where quality issues indicated in the DQ Atlas may have otherwise suggested to exclude it. DQ Atlas indicated that the proportion of service users in inpatient claims in Georgia was very high. This is because the IP file includes some OT services, but this would not impact our ability to identify disease episodes.

*Unexpected outcomes.* Despite passing all data quality checks, we also excluded Arkansas and South Carolina from our analysis due to implausibly low disease incidence rates that were quite different from bordering states.^[[3]](#footnote-4)^

## **Table A4. Diagnosis codes used to identify pneumococcal disease**

| **Condition** | **Categories** | **ICD-9 diagnoses** | | **ICD-10 diagnosis** | | **Descriptions** |
| --- | --- | --- | --- | --- | --- | --- |
| **Invasive pneumococcal disease (mapped to IPD)** | | | | | | |
| Bacteremia/ septicemia | Pneumococcal  specific | 038.2 | | A40.3 | | Pneumococcal septicemia |
|  |  | 038.0+041.2 | | A40.9+B95.3 | | Streptococcal septicemia +  Pneumococcal infection |
|  |  | 038.9+041.2 | | A41.9+B95.3 | | Unspecified septicemia +  Pneumococcal infection |
|  |  | 790.7+041.2 | | R78.81+B95.3 | | Bacteremia + Pneumococcal infection |
|  |  | 038.9+041.2 | | A41.9+B95.3 | | Unspecified septicemia +  Pneumococcal infection |
| Meningitis | Pneumococcal specific | 320.1 | | G00.1 | | Pneumococcal meningitis |
|  |  | 320.2+041.2 | | G00.2+B95.3 | | Streptococcal meningitis+ Pneumococcal infection |
|  |  | 320.9 +041.2 | | G00.9+B95.3 | | Bacterial meningitis, unspecified + Pneumococcal infection |
|  |  | 322.9 +041.2 | | G03.9+B95.3 | | Meningitis, unspecified + Pneumococcal infection |
| Bacteremic pneumonia | Pneumococcal specific | 510.x+041.2 | | J86.x+B95.3 | | Empyema + Pneumococcal infection |
|  |  | 513.0+041.2 | | J85.1+B95.3 | | Abscess of lung +  Pneumococcal infection |
|  |  | 038.2 | + one code from all-cause pneumonia | A40.3 | + one code from all-cause pneumonia | One code from the list of pneumococcal specific septicemia and one code from the list of pneumonia |
|  |  | 038.0+041.2 |  | A40.9+B95.3 |  |  |
|  |  | 038.9+041.2 |  | A41.9+B95.3 |  |  |
|  |  | 790.7+041.2 |  | R78.81+B95.3 |  |  |
|  |  | 038.0 | + 481 | A40.9 | + J13 |  |
|  |  | 038.9 |  | A41.9 |  |  |
|  |  | 790.7 |  | R78.81 |  |  |
| Other IPD | Pneumococcal specific |  | | M00.1x | | Pneumococcal arthritis |
|  |  | 567.1 | | K65.8+B95.3 | | Pneumococcal peritonitis |
|  |  | 420.9x+041.2 | | I30.1+B95.3 | | Infective pericarditis + Pneumococcal infection |
|  |  | 421.0+041.2 | | I33.0+B95.3 | | Acute and subacute bacterial endocarditis + Pneumococcal infection |
|  |  | 421.1/421.9 + 041.2 | | I33.9 +B95.3 | | Acute and subacute endocarditis, unspecified+ Pneumococcal infection |
|  |  | 567.23+041.2 | | K65.2+B95.3 | | Spontaneous bacterial peritonitis+ Pneumococcal infection |
|  |  | 730.0x, 730.2x +041.2 | | (M86.1x/M86.2x/M86.9) AND B95.3 | | Acute or unspecified osteomyelitis+ Pneumococcal infection |
|  |  | 711.0x/711.9x +041.2 | | (M00.0x/M00.2x/M00.8x/M00.9) AND B95.3 | | Pyogenic/unspecified arthritis+ Pneumococcal infection |
| **Noninvasive pneumococcal disease (mapped to pneumococcal pneumonia, ACP, and AOM)** | | | | | | |
| Pneumonia | Pneumococcal specific | 481 | | J13 | | Pneumococcal pneumonia |
|  |  | 482.9+041.2 | | J15.9 AND B95.3 | | Bacterial pneumonia, unspecified+ Pneumococcal infection |
|  |  | 485+041.2 | | (J18.0/J18.1) AND B95.3 | | Bronchopneumonia, organism unspecified+ Pneumococcal infection |
|  |  | 486+041.2 | | (J18.8/J18.9) AND B95.3 | | Pneumonia organism unspecified+ Pneumococcal infection |
| Pneumonia | All-cause pneumonia | 480.x (480.0-480.3, 480.8, 480.9) | | J12.x (J12.0, J12.1, J12.2, J12.3, J12.81, J12.89, J12.9) | | Viral pneumonia |
|  |  | 481 | | J13, J18.1 | | Pneumococcal pneumonia |
|  |  | 482.x | | A48.1, J14, J15.0, J15.1, J15.2x (J15.20, J15.211, J15.212, J15.29), J15.3, J15.4, J15.5, J15.6, J15.8, J15.9 | | Other bacterial pneumonia |
|  |  | 483.x (483.0, 483.1, 483.8) | | J15.7, J16.x (J16.0, J16.8) | | Pneumonia due to other specified organism |
|  |  | 484.x (484.1, 484.3, 484.5-484.8) | | A22.1, A37.X1, B25.0, B44.0, J17 | | Pneumonia in infectious diseases classified elsewhere |
|  |  | 485 | | J18.0 | | Bronchopneumonia, organism unspecified |
|  |  | 486 | | J18.2, J18.8, J18.9 | | Pneumonia, organism unspecified |
|  |  | 487.0 | | J09.X1, J10.0x (J10.00, J10.01, J10.08), J11.0x (J11.00, J11.08) | | Influenza with pneumonia |
| Acute Otitis Media | Acute Otitis Media | 382.x | | H66.xxx, H67.x | | Acute suppurative otitis media, suppurative and unspecified OM |

## **Table A5. Moran’s I to assess spatial autocorrelation in county-level disease incidence rates among Medicaid children**

| **Measure** | **Coefficient** | **Z** | **Pr > \|Z\|** |
| --- | --- | --- | --- |
| All-cause pneumonia | 0.251 | 17.47 | <0.0001 |
| Acute otitis media | 0.541 | 36.80 | <0.0001 |
| Invasive pneumococcal disease | 0.0127 | 0.93 | 0.35 |

## **Table A6. Study cohort construction – number of Medicaid children at each stage of cohort creation**

| **Cohort construction step** | **Number of children** |
| --- | --- |
| All children (aged 18 and under) in enrollment files, 2017-2019 | 50,027,834 |
| After dropping children who are dually eligible or in long-term care | 50,012,611 |
| After applying eligibility criteria | 45,459,960 |
| After dropping states with data quality concerns and counties without MHSVI data | 38,106,592 |

In Tables A7 and A8, we examined the relationship (unadjusted and regression-adjusted, respectively) between area-level vulnerability and AOM, ACP, and IPD incidence within demographic subgroups based on age groups and race and ethnicity. Because disease risk for younger children is higher than for older children, we partitioned each child’s study period such that their disease episodes count towards the subgroup during the years they were within the corresponding age range. For example, if a child was born in January 2017 and observable during the entire study period, we would include one period from January through December 2017 for the subgroup analyses of infants less than one year old, then a second period from January through December 2018 for the analysis of one-year-olds, and then a third period from January through December 2019 for the analysis of children aged 2-4.

## **Table A7. Unadjusted ACP, AOM, and IPD incidence among children in Medicaid cohort, by group and county-level social vulnerability quintile, overall MHSVI (2017–2019)**

| **Group** | **Number of children** | **Incidence per 100,000 PY** | **Unadjusted incidence per 100,000 PY by MHSVI quintile** | | | | | ***p*-value (joint test for all quintiles)^a^** |
| --- | --- | --- | --- | --- | --- | --- | --- | --- |
|  |  |  | **Q1** | **Q2** | **Q3** | **Q4** | **Q5** |  |
| **ACP** | | | | | | | | |
| **Overall** | 38,106,592 | 1,767 | 1,888 | 1,928 | 1,859 | 1,756 | 1,688 | <0.0001 |
| **Age group (years)^b^** | | | | | | | | |
| < 1 | 4,463,679 | 4,046 | 4,129 | 3,883 | 4,038 | 3,836 | 4,195 | <0.0001 |
| 1 | 2,345,947 | 3,918 | 4,045 | 4,008 | 4,024 | 3,820 | 3,897 | <0.0001 |
| 2–4 | 6,398,734 | 2,915 | 2,949 | 3,150 | 3,090 | 2,920 | 2,783 | <0.0001 |
| 5–18 | 25,870,005 | 1,074 | 1,228 | 1,272 | 1,168 | 1,073 | 977 | <0.0001 |
| **Race and ethnicity** | | | | | | | | |
| White, non-Hispanic | 11,668,956 | 1,858 | 1,828 | 1,930 | 1,889 | 1,873 | 1,790 | <0.0001 |
| Black, non-Hispanic | 6,308,212 | 1,515 | 1,812 | 1,634 | 1,659 | 1,445 | 1,493 | <0.0001 |
| Hispanic | 9,678,079 | 1,538 | 1,619 | 1,764 | 1,598 | 1,610 | 1,489 | <0.0001 |
| Asian | 1,292,228 | 1,935 | 1,440 | 2,046 | 1,745 | 2,108 | 1,947 | <0.0001 |
| Other | 916,553 | 2,099 | 2,162 | 1,810 | 1,968 | 2,075 | 2,225 | <0.0001 |
| **AOM** | | | | | | | | |
| **Overall** | 38,106,592 | 16,486 | 20,653 | 18,541 | 17,240 | 16,430 | 15,432 | <0.0001 |
| **Age group (years)^b^** | | | | | | | | |
| < 1 | 4,463,679 | 49,991 | 63,750 | 54,271 | 51,950 | 50,224 | 47,167 | <0.0001 |
| 1 | 2,345,947 | 50,593 | 63,870 | 57,057 | 52,831 | 50,798 | 47,186 | <0.0001 |
| 2–4 | 6,398,734 | 28,112 | 35,025 | 32,246 | 29,766 | 27,584 | 26,266 | <0.0001 |
| 5–18 | 25,870,005 | 7,370 | 9,297 | 8,604 | 7,832 | 7,151 | 6,865 | <0.0001 |
| **Race and ethnicity** | | | | | | | | |
| White, non-Hispanic | 11,668,956 | 20,224 | 19,778 | 19,997 | 20,405 | 20,837 | 19,815 | <0.0001 |
| Black, non-Hispanic | 6,308,212 | 11,048 | 13,723 | 12,133 | 11,676 | 11,633 | 10,341 | <0.0001 |
| Hispanic | 9,678,079 | 14,194 | 18,570 | 16,716 | 13,522 | 13,978 | 14,147 | <0.0001 |
| Asian | 1,292,228 | 8,957 | 13,000 | 10,708 | 7,791 | 9,331 | 8,877 | <0.0001 |
| Other | 916,553 | 16,736 | 18,056 | 16,063 | 14,762 | 16,242 | 17,958 | <0.0001 |
| **IPD** | | | | | | | | |
| **Overall** | 38,106,592 | 3.3 | 3.9 | 3.2 | 3.1 | 3.3 | 3.4 | 0.25 |
| **Age group (years)^b^** | | | | | | | | |
| < 1 | 4,463,679 | 11.3 | 13.9 | 10.5 | 12.1 | 10.2 | 11.6 | 0.38 |
| 1 | 2,345,947 | 7.6 | 9.5 | 5.7 | 5.3 | 8.9 | 8.1 | 0.01 |
| 2–4 | 6,398,734 | 4.3 | 4.2 | 6.0 | 3.5 | 3.9 | 4.4 | 0.007 |
| 5–18 | 25,870,005 | 1.9 | 2.4 | 1.6 | 1.9 | 1.9 | 1.9 | 0.28 |
| **Race and ethnicity** | | | | | | | | |
| White, non-Hispanic | 11,668,956 | 3.0 | 4.1 | 2.9 | 2.8 | 3.3 | 2.8 | 0.07 |
| Black, non-Hispanic | 6,308,212 | 3.5 | 1.8 | 3.0 | 2.9 | 3.5 | 3.8 | 0.16 |
| Hispanic | 9,678,079 | 2.5 | 3.2 | 1.9 | 2.3 | 2.8 | 2.5 | 0.40 |
| Asian | 1,292,228 | 2.7 | 7.6 | 2.9 | 2.3 | 2.4 | 2.9 | 0.40 |
| Other | 916,553 | 4.9 | 1.7 | 3.3 | 3.8 | 3.2 | 6.9 | 0.007 |

ACP = all-cause pneumonia; AOM = acute otitis media; IPD = invasive pneumococcal disease; MHSVI = Minority Health Social Vulnerability Index; PY = person-years; Q1 is least vulnerable quintile; Q5 is most vulnerable quintile.

^a^ The joint test evaluates whether the regression coefficients for all quintile categories are statistically significant from zero together, which assesses whether vulnerability (as divided into quintiles) affects disease incidence overall.

^b^ The sum of children across age groups is larger than the total number of children in the cohort because we partitioned each child’s study period such that their outcomes count towards the age group when they were within the corresponding age range.

## **Table A8. Difference in ACP, AOM, and IPD incidence among children in the Medicaid cohort residing in the most versus least socially vulnerable counties (2017–2019)**

| **Group** | **Unadjusted results** | | | | | **Regression-adjusted results** | | | | |
| --- | --- | --- | --- | --- | --- | --- | --- | --- | --- | --- |
|  | **Number of children** | **IRR (Q5/Q1)** | **95% CI around IRR** | **IRR  *p*-value** | **AIC** | **Number of children** | **IRR  (Q5/Q1)** | **95% CI around IRR** | **IRR  *p*-value** | **AIC** |
| **ACP** | | | | | | | | | | |
| **Overall** | 38,106,592 | 0.89 | (0.886, 0.902) | <0.0001 | 13,484,728 | 38,093,362 | 0.97 | (0.957, 0.975) | <0.0001 | 12,949,834 |
| **Age^a^** | | | | | | | | | | |
| < 1 | 4,463,679 | 1.02 | (0.993, 1.039) | 0.17 | 2,050,791 | 4,452,021 | 1.17 | (1.141, 1.196) | <0.0001 | 2,039,619 |
| 1 | 2,345,947 | 0.96 | (0.938, 0.989) | 0.005 | 1,380,974 | 2,345,152 | 1.05 | (1.023, 1.080) | 0.0003 | 1,377,430 |
| 2–4 | 6,398,734 | 0.94 | (0.928, 0.961) | <0.0001 | 3,308,407 | 6,398,422 | 0.98 | (0.963, 0.999) | 0.04 | 3,305,891 |
| 5–18 | 25,870,005 | 0.80 | (0.784, 0.806) | <0.0001 | 6,301,179 | 25,869,518 | 0.87 | (0.859, 0.883) | <0.0001 | 6,290,256 |
| **Race and ethnicity** | | | | | | | | | | |
| White, non-Hispanic | 11,668,956 | 0.98 | (0.967, 0.992) | 0.001 | 4,239,142 | 11,664,065 | 0.95 | (0.935, 0.960) | <0.0001 | 4,113,077 |
| Black, non-Hispanic | 6,308,212 | 0.82 | (0.788, 0.861) | <0.0001 | 2,014,371 | 6,303,386 | 0.82 | (0.787, 0.860) | <0.0001 | 1,914,595 |
| Hispanic | 9,678,079 | 0.92 | (0.894, 0.947) | <0.0001 | 3,151,486 | 9,677,359 | 0.88 | (0.855, 0.909) | <0.0001 | 3,018,301 |
| Asian | 1,292,228 | 1.35 | (1.258, 1.454) | <0.0001 | 487,311 | 1,292,061 | 1.27 | (1.181, 1.370) | <0.0001 | 469,730 |
| Other | 916,553 | 1.03 | (0.973, 1.088) | 0.31 | 375,676 | 916,336 | 1.05 | (0.989, 1.107) | 0.12 | 353,067 |
| **AOM** | | | | | | | | | | |
| **Overall** | 38,106,592 | 0.75 | (0.745, 0.749) | <0.0001 | 71,310,938 | 38,093,362 | 0.93 | (0.930, 0.936) | <0.0001 | 60,686,577 |
| **Age^a^** | | | | | | | | | | |
| < 1 | 4,463,679 | 0.74 | (0.736, 0.744) | <0.0001 | 11,580,240 | 4,452,021 | 0.92 | (0.912, 0.923) | <0.0001 | 11,427,068 |
| 1 | 2,345,947 | 0.74 | (0.734, 0.744) | <0.0001 | 7,741,573 | 2,345,152 | 0.91 | (0.908, 0.921) | <0.0001 | 7,635,885 |
| 2–4 | 6,398,734 | 0.75 | (0.746, 0.754) | <0.0001 | 15,821,970 | 6,398,422 | 0.93 | (0.922, 0.931) | <0.0001 | 15,625,601 |
| 5–18 | 25,870,005 | 0.74 | (0.735, 0.742) | <0.0001 | 26,885,051 | 25,869,518 | 0.97 | (0.964, 0.974) | <0.0001 | 26,460,098 |
| **Race and ethnicity** | | | | | | | | | | |
| White, non-Hispanic | 11,668,956 | 1.00 | (0.998, 1.006) | 0.35 | 24,736,471 | 11,664,065 | 0.99 | (0.989, 0.996) | 0.0001 | 21,350,298 |
| Black, non-Hispanic | 6,308,212 | 0.75 | (0.742, 0.766) | <0.0001 | 9,254,991 | 6,303,386 | 0.75 | (0.737, 0.761) | <0.0001 | 7,482,109 |
| Hispanic | 9,678,079 | 0.76 | (0.755, 0.768) | <0.0001 | 16,340,627 | 9,677,359 | 0.85 | (0.847, 0.862) | <0.0001 | 14,431,678 |
| Asian | 1,292,228 | 0.68 | (0.666, 0.700) | <0.0001 | 1,571,334 | 1,292,061 | 0.79 | (0.772, 0.812) | <0.0001 | 1,388,626 |
| Other | 916,553 | 0.99 | (0.976, 1.014) | 0.58 | 1,746,573 | 916,336 | 1.05 | (1.029, 1.070) | <0.0001 | 1,472,812 |
| **IPD** | | | | | | | | | | |
| **Overall** | 38,106,592 | 0.87 | (0.709, 1.058) | 0.16 | 63,490 | 38,093,362 | 0.86 | (0.701, 1.057) | 0.15 | 61,898 |
| **Age^a^** | | | | | | | | | | |
| < 1 | 4,463,679 | 0.83 | (0.562, 1.232) | 0.36 | 14,784 | 4,452,021 | 0.86 | (0.576, 1.294) | 0.48 | 14,715 |
| 1 | 2,345,947 | 0.85 | (0.492, 1.460) | 0.55 | 7,373 | 2,345,152 | 0.94 | (0.537, 1.654) | 0.84 | 7,369 |
| 2–4 | 6,398,734 | 1.04 | (0.652, 1.651) | 0.88 | 13,331 | 6,398,422 | 0.95 | (0.593, 1.534) | 0.85 | 13,319 |
| 5–18 | 25,870,005 | 0.81 | (0.592, 1.096) | 0.17 | 26,692 | 25,869,518 | 0.80 | (0.581, 1.097) | 0.16 | 26,611 |
| **Race and ethnicity** | | | | | | | | | | |
| White, non-Hispanic | 11,668,956 | 0.69 | (0.525, 0.917) | 0.010 | 17,924 | 11,664,065 | 0.66 | (0.495, 0.873) | 0.004 | 17,563 |
| Black, non-Hispanic | 6,308,212 | 2.14 | (0.532, 8.592) | 0.28 | 11,287 | 6,303,386 | 1.84 | (0.459, 7.410) | 0.39 | 10,952 |
| Hispanic | 9,678,079 | 0.79 | (0.410, 1.539) | 0.50 | 12,589 | 9,677,359 | 0.73 | (0.366, 1.455) | 0.02 | 12,313 |
| Asian | 1,292,228 | 0.37 | (0.133, 1.054) | 0.06 | 1,746 | 1,292,061 | 0.27 | (0.092, 0.784) | 0.37 | 1,728 |
| Other | 916,553 | 4.18 | (0.580, 30.126) | 0.16 | 2,209 | 916,336 | 4.45 | (0.611, 32.351) | 0.14 | 2,173 |

ACP = all-cause pneumonia; AIC = Akaike information criterion; AOM = acute otitis media; CI = confidence interval; IPD = invasive pneumococcal disease; IRR = incidence rate ratio; MHSVI = Minority Health Social Vulnerability Index; Q1 = least vulnerabe quintile; Q5 = most vulnerable quintile.

^a^ The sum of children across age groups is larger than the total number of children in the cohort because we partitioned each child’s study period such that their outcomes count towards the age group when they were within the corresponding age range.

## **Table A9. Unadjusted ACP, AOM, and IPD incidence among children in the Medicaid cohort, by MHSVI quintile and theme (2017–2019)**

| **Theme** | **Unadjusted incidence per 100,000 PY by MHSVI quintile** | | | | | **IRR (Q5/Q1)** | **IRR *p*-value** |
| --- | --- | --- | --- | --- | --- | --- | --- |
|  | **Q1** | **Q2** | **Q3** | **Q4** | **Q5** |  |  |
| **ACP** | | | | | | | |
| Overall MHSVI | 1,888 | 1,928 | 1,859 | 1,756 | 1,688 | 0.89 | <0.0001 |
| Socioeconomic Status | 1,888 | 1,744 | 1,713 | 1,821 | 1,691 | 0.90 | 0.02 |
| Household Composition and Disability | 1,781 | 1,731 | 1,719 | 1,740 | 1,982 | 1.11 | 0.02 |
| Minority Status and Language | 1,965 | 2,057 | 1,938 | 1,908 | 1,710 | 0.87 | <0.0001 |
| Housing Type and Transportation | 1,834 | 1,851 | 1,775 | 1,718 | 1,769 | 0.96 | 0.42 |
| Health Care Infrastructure and Access | 1,831 | 1,811 | 1,664 | 1,794 | 1,762 | 0.96 | 0.25 |
| Medical Vulnerability | 1,748 | 1,620 | 1,774 | 1,969 | 2,047 | 1.17 | <0.0001 |
| **AOM** | | | | | | | |
| Overall MHSVI | 20,653 | 18,541 | 17,240 | 16,430 | 15,432 | 0.75 | <0.0001 |
| Socioeconomic Status | 16,092 | 16,614 | 16,414 | 16,117 | 17,357 | 1.08 | 0.26 |
| Household Composition and Disability | 13,534 | 17,241 | 17,376 | 18,516 | 21,459 | 1.59 | <0.0001 |
| Minority Status and Language | 24,343 | 23,766 | 22,840 | 20,890 | 14,684 | 0.60 | <0.0001 |
| Housing Type and Transportation | 20,099 | 19,382 | 17,809 | 17,001 | 14,075 | 0.70 | <0.0001 |
| Health Care Infrastructure and Access | 16,898 | 15,068 | 15,850 | 19,610 | 20,519 | 1.21 | <0.0001 |
| Medical Vulnerability | 13,913 | 16,428 | 18,684 | 20,872 | 23,405 | 1.68 | <0.0001 |
| **IPD** | | | | | | | |
| Overall MHSVI | 3.9 | 3.2 | 3.1 | 3.3 | 3.4 | 0.87 | 0.16 |
| Socioeconomic Status | 3.2 | 3.6 | 3.2 | 3.2 | 3.3 | 1.04 | 0.79 |
| Household Composition and Disability | 3.1 | 3.4 | 3.7 | 3.4 | 3.0 | 0.97 | 0.77 |
| Minority Status and Language | 2.3 | 3.4 | 2.9 | 3.8 | 3.3 | 1.45 | 0.01 |
| Housing Type and Transportation | 3.4 | 3.0 | 3.3 | 3.4 | 3.3 | 0.98 | 0.94 |
| Health Care Infrastructure and Access | 3.7 | 3.3 | 3.1 | 3.2 | 2.9 | 0.78 | 0.05 |
| Medical Vulnerability | 3.3 | 3.4 | 3.3 | 3.9 | 2.7 | 0.82 | 0.07 |

ACP = all-cause pneumonia; AOM = acute otitis media; IPD = invasive pneumococcal disease; IRR = incidence rate ratio; MHSVI = Minority Health Social Vulnerability Index; PY = person-years; Q1 is least vulnerabe quintile; Q5 is most vulnerable quintile

1. https://www.medicaid.gov/dq-atlas/welcome [↑](#footnote-ref-2)
2. The OT file includes professional and institutional claims for a wide range of services, including physician services and outpatient hospital services, that do not fit into the inpatient, long-term care, and pharmacy claims files. [↑](#footnote-ref-3)
3. Colorado and New Mexico also had unexpectedly low disease incidence, but since this could be due to high vaccination rates, we kept them in our analysis. [↑](#footnote-ref-4)
